# Proteomics integrated with metabolomics: analysis of the internal causes of nutrient changes in alfalfa at different growth stages

**DOI:** 10.1186/s12870-018-1291-8

**Published:** 2018-05-04

**Authors:** Wenqiang Fan, Gentu Ge, Yinghao Liu, Wei Wang, Liying Liu, Yushan Jia

**Affiliations:** College of Grassland Resources and Environment, Key Laboratory of Forage Cultivation, Processing and High-Efficiency Utilization of the Ministry of Agriculture and Key Laboratory of Grassland Resources of the Ministry of Education, Inner Mongolia Agricultural University, Hohhot, 010011 China

**Keywords:** Alfalfa, Growth stages, Nutritional quality, Metabolomics, Proteomics

## Abstract

**Background:**

Alfalfa (*Medicago sativa* L.) is one of the most important forage resources in the world due to its high nutritive value. However, its nutritional quality decreases during the transition from budding to flowering. Previous research revealed a decreased crude protein content and increased fibre content in alfalfa forage harvested at later maturity stages, leading to a reduction in nutritional quality. However, the reasons for this phenomenon have not been explained at the molecular level.

**Results:**

In this study, leaves from the WL319HQ alfalfa cultivar were harvested at two developmental stages (budding and mid-flowering). The leaves were used to test the variable expression of proteins and metabolites during these stages. TMT-based quantitative proteomics and LC-MS/MS-based untargeted metabolomics methods were employed in this study. A total of 415 proteins and 49 metabolites showed at least a 1.2-fold difference in abundance during these stages. Most of the differentially expressed proteins and metabolites were involved in metabolic processes, including carbohydrate metabolism, starch and sucrose metabolism, phenylpropanoid biosynthesis, and biosynthesis of amino acids. Alfalfa leaves in mid-flowering contain less crude protein due to the decrease in L-glutamic acid content. Carbohydrate metabolism provides the raw material for the synthesis of hemicellulose, resulting in an increase in the hemicellulose content of the alfalfa leaves, leading to an increase in the NDF content. In addition, the increase in L-phenylalanine content could have provided the conditions necessary for lignin synthesis. These are the main factors leading to reductions in alfalfa relative feed value (RFV) and quality.

**Conclusions:**

This study used joint proteomic and metabolomic analyses to elucidate the relationship between the reduction in the nutritional value of alfalfa and complex biological processes. This provides a theoretical basis for producing high-quality alfalfa hay and sets the stage for further research.

**Electronic supplementary material:**

The online version of this article (10.1186/s12870-018-1291-8) contains supplementary material, which is available to authorized users.

## Background

Alfalfa (*Medicago sativa* L.) is a well-known forage crop that has been cultivated since antiquity. It is the most widely grown leguminous crop in the world and exhibits high protein, amino acid, vitamin and mineral contents [1, 2]. Hay making is the most important method of alfalfa utilization and conservation [3]. Alfalfa is referred to as the “queen of forage” because of the high nutritive value of its leaves and the fact that animals will readily eat it both when it is green and as hay [4]. However, the nutritive value of alfalfa hay is influenced by many factors, including the harvest period [3, 5, 6]. Alfalfa harvested at the budding stage has a greater leaf yield than stem yield, but the early flower leaf and stem yields are nearly the same. At the late flowering stage, the stem fraction of forage is greater than that of the leaves [7]. Many researchers have also reported decreased crude protein (CP) and increased fibre contents in alfalfa forage harvested at advancing maturity stages [8]. Cutting alfalfa at the optimum growth stage can improve both hay yield and quality [9]. Harvesting at the early flower stage is currently thought to result in high yields and nutrient concentrations in alfalfa forage. However, this is difficult to achieve in China, where alfalfa is always harvested from the budding stage until the mid-flowering stage due to the temperate semi-arid continental climate and the lack of mechanical equipment. Many metabolites and nutrients, especially proteins, may decrease during this time, resulting in a reduction in the relative feed value (RFV), which is a widely accepted forage quality index [10], indicating a large impact on the production of high-quality alfalfa hay and the stable and sustainable development of animal husbandry.

To determine exactly what happens to the nutrients during these changes, we considered the use of omics methods. Metabolomics and proteomics offer an effective approach for identifying metabolites, proteins and associated pathways that are crucial for understanding the nutrient contents of alfalfa and the mechanisms underlying nutrient metabolite changes during different growth stages. Most of the current research on alfalfa focuses on using proteomics to identify stress resistance genes [11]. However, few studies have focused on the nutrient metabolism and internal factors that affect the quality of alfalfa hay during alfalfa development. Recent advances in metabolomics and proteomics technologies are greatly expediting the identification and characterization of natural products and their associated metabolites [12, 13]. In this regard, integrated metabolo-proteomics [11, 14], using high-resolution nano-liquid chromatography coupled to tandem mass spectrometry (nanoLC-MS/MS) [12], is a good method for identifying metabolites or proteins that may lead to reductions in the nutritional quality of alfalfa.

In this study, we conducted an integrated untargeted metabolomics and tandem mass tag (TMT)-based proteomic analysis. Our objective was to gain a comprehensive understanding of the nutrient changes in alfalfa and the causes of these changes from the budding to flowering stages. We also wanted to identify key regulatory pathways and proteins that contribute to changes in metabolites and nutrient contents in alfalfa.

## Methods

### Plant material

**Fig. 1:**
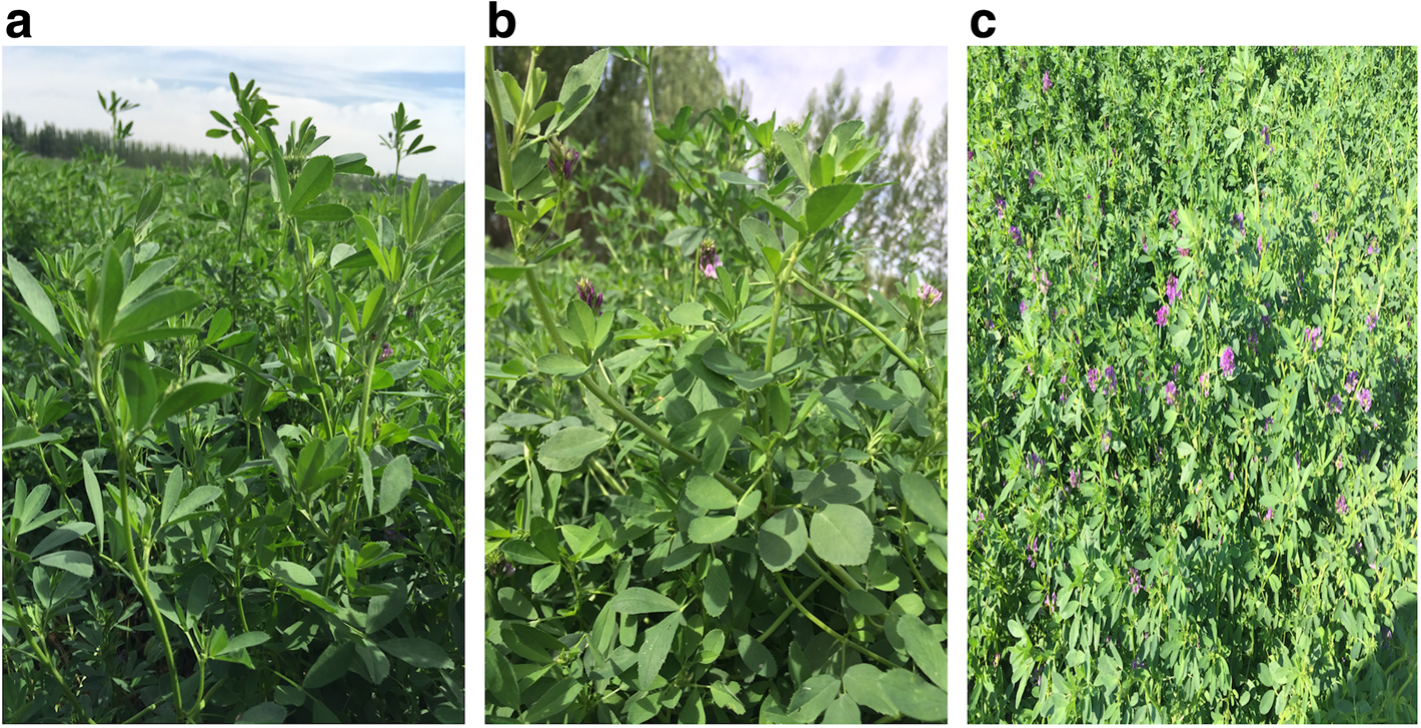
Alfalfa at different growth stages. Alfalfa at different growth stages. **a** Alfalfa in the budding stage. **b** Alfalfa in the early flowering stage. **c** Alfalfa in the mid-flowering stage

Alfalfa (*Medicago sativa* L.) was grown in a field at the Inner Mongolia Agricultural University field experimental station in Baotou (40.60°N, 109.75°E), Inner Mongolia, China, in 2015. We had permission to use this field for our study, and all experiments were conducted in accordance with local legislation. Leaves were selected as samples. The harvest times corresponded to three distinct stages: budding (large area of alfalfa budding), early flowering (10% flowering) and mid-flowering (45% flowering) (Fig. 1). Leaf samples of 150 g were collected from alfalfa at each of these three stages and then dried at 65 °C for nutrition quality analysis. At the budding stage and mid-flowering stage, 50 g leaf samples were collected from alfalfa and immediately frozen in liquid nitrogen, then stored at − 80 °C for protein and metabolite extraction. The treatments were repeated in triplicate for proteomics and nutrition analyses and were repeated nine times for metabolomics analysis.

### Nutrition quality analysis

Crude protein (CP) was analysed using the AOAC [15] method and calculated as N × 6.25. Fibre was measured as described by Van Soest et al. [16], with the samples being sequentially digested using an Ankom 220 Fiber Analyzer (Ankom Technology, Fairport, NY, USA) in accordance with the recommendations for neutral detergent fibre (NDF) and acid detergent fibre (ADF) analyses. The relative feed value (RFV) index was estimated as the digestible dry matter (DDM) content of the samples based on ADF values. First, the dry matter intake (DMI) potential (as a percentage of body weight, BW) was calculated from NDF values, and the index was then calculated as DDM multiplied by DMI as a % of BW and divided by 1.29 [17].

$$ {\displaystyle \begin{array}{l}\mathrm{DDM}=88.9-\left(0.779\times \%\mathrm{ADF}\right).\\ {}\mathrm{DMI}=120/\left(\%\mathrm{NDF}\right)\\ {}\mathrm{RFV}=\left(\mathrm{DDM}\times \mathrm{DMI}\right)/1.29\end{array}} $$

### TMT analysis methods

#### Protein extraction, digestion, TMT labelling and strong cation exchange chromatography

Proteins were extracted using TCA/acetone precipitation and the SDT (4% SDS, 100 mM DTT, 150 mM Tris-HCl pH 8.0) lysis method [18]. Samples of approximately 200 mg were frozen in liquid nitrogen and ground with a mortar and pestle before being transferred to a 10–15 ml centrifuge tube. Next, five volumes of TCA/acetone (1:9) were added to the powder, followed by vortexing. The mixture was then precipitated at − 20 °C for 4 h and centrifuged (6000 g, 4 °C, 40 min), and the supernatant was discarded. Pre-cooling acetone was subsequently added, and washing was performed three times. The precipitate was then air dried, and 30 volumes of SDT buffer was added to 20 mg of the powder, followed by mixing and boiling for 5 min. Thereafter, the lysate was sonicated (80 W, 10 s, 10 times) and boiled for 15 min. After centrifugation (14,000 g, 4 °C, 40 min), the supernatant was filtered with 0.22 μm filters. The filtrate was then quantified using a BCA Protein Assay Kit (Bio-Rad, USA), and the sample was stored at − 80 °C. Protein digestion and peptide quantification were performed according to the FASP procedure described by Wisniewski et al. [19]. The digested peptides of each sample were desalted in C18 Cartridges (Empore™ SPE Cartridges C18, standard density, bed I.D. 7 mm, volume 3 ml, Sigma), concentrated via vacuum centrifugation and reconstituted in 40 μl of 0.1% (*v*/v) formic acid. The peptide content was estimated based on the UV light spectral density at 280 nm, with an extinction coefficient of 1.1 for a 0.1% (g/l) solution, which was calculated based on the frequency of tryptophan and tyrosine in vertebrate proteins.

For labelling, each TMT reagent was dissolved in 70 μl of ethanol and added to the respective peptide mixture. For each sample, 100 μg of the peptide mixture was labelled using the 6-plex TMT reagent according to the manufacturer’s instructions (TMT Mass Tagging Kits and Reagents. Thermo Fisher Scientific). The samples were labelled as (XLQ-1)-126, (XLQ-2)-127, (XLQ-3)-128, (ZHQ-1)-129, (ZHQ-2)-130 and (ZHQ-3)-131 and then multiplexed and vacuum dried.

A Pierce high-pH reverse-phase fractionation kit (Thermo Scientific) was employed to fractionate the TMT-labelled digested samples into ten fractions via increasing acetonitrile step-gradient elution according to the manufacturer’s instructions.

#### Mass spectrometry

Each fraction was injected for nanoLC-MS/MS analysis. The peptide mixture was loaded onto a reverse-phase trap column (Thermo Scientific Acclaim PepMap100, 100 μm × 2 cm, nanoViper C18) connected to a C18 reverse-phase analytical column (Thermo Scientific Easy Column, 10 cm long, 75 μm inner diameter, 3 μm resin) in buffer A (0.1% formic acid) and separated with a linear gradient of buffer B (84% acetonitrile and 0.1% formic acid) at a flow rate of 300 nl/min, controlled by IntelliFlow technology. The analysis gradient was a 1-h gradient consisting of 0–50% buffer B for 50 min, 50–100% buffer B for 5 min, and holding in 100% buffer B for 5 min.

LC-MS/MS analysis was performed on a Q Exactive mass spectrometer (Thermo Scientific) coupled to an Easy nLC system (Proxeon Biosystems, now Thermo Fisher Scientific) for 60 min. The mass spectrometer was operated in positive ion mode. MS data were acquired using a data-dependent top 10 method, dynamically choosing the most abundant precursor ions from the survey scan (300–1800 m/z) for HCD fragmentation. The automatic gain control (AGC) target was set to 3e6 and the maximum injection time to 10 ms. The duration of dynamic exclusion was 40.0 s. Survey scans were acquired at a resolution of 70,000 at m/z 200; the resolution for HCD spectra was set to 17,500 at m/z 200; and the isolation width was 2 m/z. The normalized collision energy was 30 eV. The underfill ratio, which specifies the minimum percentage of the target value likely to be reached at the maximum fill time, was defined as 0.1%. The instrument was run with peptide recognition mode enabled.

#### Database search and data analysis

MS/MS spectra were searched using the MASCOT engine (Matrix Science, London, UK; version 2.2) embedded into Proteome Discoverer 1.4 (Thermo Fisher Scientific Inc. Xcalibur Proteome Discoverer Version 1.4 User Guide). The acquired MS/MS spectra were automatically searched against Uniprot database (“Uniprot_Medicago_71871_20160801.fasta” downloaded from http://www.uniprot.org/ on August 1, 2016, which includes 71,871 protein sequences). The related search parameters were as follows: enzyme = trypsin; max missed cleavage = 2; fixed modifications: carbamidomethyl (C), TMT 6plex (N-term), TMT 6plex (K); variable modifications: Oxidation (M), TMT 6plex (Y); peptide mass tolerance = ± 20 ppm; fragment mass tolerance = 0.1 Da. For unique proteins with at least two unique peptides, the false discovery rate (FDR) was set to < 0.01 for the identification of both peptides and proteins. Proteome Discoverer 1.4 software was used for the quantitative analysis of the peak intensity values of the ions of peptide fragments. To designate significant changes in protein expression, a fold-change of > 1.2 or < 0.83 and a *P*-value of < 0.05 using Student’s t-test were set as cut-off values [20, 21].

### Untargeted metabolomics analysis methods

#### Metabolite extraction

Frozen samples were thawed at 4 °C and ground in liquid nitrogen. The weight of each sample from each group was approximately 80 mg. Metabolites were extracted by adding 1 ml of methanol:acetonitrile:water (2:2:1, *v*/v), followed by vortexing for 60 s. Ultrasonic crushing was performed at a low temperature, two times for 30 min each time. The samples were then centrifuged at high speed (13,000 rpm, 4 °C) for 15 min, and the supernatant was dried in a vacuum centrifuge, transferred to new tubes and stored at − 80 °C. To monitor the stability and repeatability of the instrument analysis, quality control (QC) samples were prepared by pooling 10 μl of each sample, and these samples were analysed together with the other samples. The QC samples were inserted regularly and analysed every five samples.

#### LC-MS/MS analysis

LC-MS/MS analyses were performed using a UHPLC (1290 Infinity LC, Agilent Technologies) coupled to a quadrupole time-of-flight mass spectrometer (AB Sciex TEIPLE TOF 6600).

For HILIC separation, samples were analysed using a 2.1 × 100 mm ACQUITY UPLC BEH 1.7 μm column (Waters, Ireland). In both ESI positive and negative modes, the mobile phase contained A = 25 mM ammonium acetate and 25 mM ammonium hydroxide in water and B = acetonitrile. The gradient was 85% B for 1 min, which was linearly reduced to 65% over 11 min and then to 40% over 0.1 min, where it was held for 2.9 min and then increased to 85% over 0.1 min. A 5 min re-equilibration period was employed.

The ESI source conditions were set as follows: ion source Gas (Gsa1), 60; ion source Gsa2 (Gas2), 60; curtain gas (CUR), 30; source temperature, 600 °C; ion spray voltage floating (ISVF), ± 5500 V. During MS-only acquisition, the instrument was set for acquisition over an m/z range of 60–1000 Da, and the accumulation time for TOF MS scanning was set at 0.20 s/spectrum. During auto MS/MS acquisition, the instrument was set to acquire over an m/z range of 25–1000 Da, and the accumulation time for product ion scanning was set at 0.05 s/spectra. The parameters were set as follows: collision energy (CE) fixed at 35 V with ±15 eV; declustering potential (DP), 60 V (+) and − 60 V (−); exclusion of isotopes within 4 Da; candidate ions monitored per cycle, 10.

#### Data processing and statistical data analysis

The raw MS data (wiff.scan files) were converted to MzXML files using ProteoWizard MSConvert and processed using XCMS [22–24] for feature detection, retention time correction and alignment. Metabolites were identified via accuracy mass (< 25 ppm) matching and secondary spectrogram matching (score > 0.8). The results were queried and compared with a laboratory standards database (Shanghai Applied Protein Technology Co., Ltd.)

In the extracted ion features, only the variables exhibiting more than 50% nonzero measurement values in at least one group were retained. For the multivariate statistical analysis, SIMCA-P (version 14.1, Umetrics, Umea, Sweden) was employed. After Pareto scaling, principal component analysis (PCA) and orthogonal partial least-squares discriminant analyses (OPLS-DA) were performed, and 7-fold cross-validation and response permutation testing were used to evaluate the robustness of the model. The significantly different metabolites were determined based on the combination of a statistically significant threshold of variable influence on the projection (VIP) values obtained from the OPLS-DA model and Student’s t-test (*P*-value) on the raw data, and the metabolites with a VIP > 1.0 and *P*-values < 0.05 were considered statistically significant [25].

### Bioinformatics analysis

To determine the functional classification and biological properties of the selected differentially abundant proteins, the identified protein sequences were mapped using Gene Ontology (GO) terms. For this analysis, a homology search was performed for all of the identified sequences with a localized NCBI BLAST search against the NCBInr *Medicago truncatula* database. GO annotation was performed using BLAST2GO [26]. The GO project described the roles of proteins in three functional categories: biological process (BP), cellular component (CC) and molecular function (MF). In addition, all differentially abundant proteins and metabolites were queried against the online Kyoto Encyclopedia of Genes and Genomes (KEGG, http://www.kegg.jp/) [27] and mapped to KEGG pathways. To further explore the impact of differentially expressed proteins and identify internal relationships between differentially expressed proteins, enrichment analysis was performed. Only functional categories and pathways with *P*-values < 0.05 were considered to have significant enrichment. The different metabolites and significantly different proteins identified between the mid-flowering-stage and the budding-stage alfalfa leaves were employed separately to query the integrated KEGG metabolites and proteins with an R-based software tool for omics data integration.

### Statistical analysis

All experiments were repeated with three independent biological replicates. The nutritional indicator data were analysed using one-way ANOVA with SPSS 20.0 statistical software (v 20.0, SPSS Inc. Chicago, USA). The mean differences were compared using Duncan’s multiple range t-test. Comparisons with *P* < 0.05 were considered significantly significant, and the data are presented as the mean ± standard errors of at least three independent replicates.

## Results

### Alfalfa leaf nutrient content analysis

To assess the composition of the leaves at different stages, the main nutritional indexes were determined and recorded (Table 1). CP analysis showed that the CP content of alfalfa leaves was highest in the budding stage, at 29.00, and lowest in the mid-flowering stage, at 26.84. These results revealed that the CP content of leaves continues to decrease from the budding to mid-flowering stage. In contrast, the NDF content at the mid-flowering stage was 22.43, which was significantly higher than at the early flowering and budding stages (*P* < 0.05). There was no significant difference in ADF content between the budding and early flowering stages, which was significantly lower than in the mid-flowering stage (*P* > 0.05). Fibre analysis indicated that the NDF and ADF of alfalfa leaves gradually increased. Thus, these factors caused the alfalfa RFV to decline from the budding to mid-flowering stages.

**Table 1:** Nutrients and relative feed value of alfalfa in different growth periods

Growth period	CP (%DM)	NDF (%DM)	ADF (%DM)	RFV
Budding stage	29.00 ± 0.08^a^	19.36 ± 0.06^a^	12.14 ± 0.02^a^	381.72
Early flowering	27.69 ± 0.09^b^	21.58 ± 0.06^b^	12.16 ± 0.01^a^	342.38
Mid-flowering	26.84 ± 0.10^c^	22.43 ± 0.05^c^	12.21 ± 0.02^b^	329.25

^a, b, c^Means ± SD within columns with different superscripted letters indicating significant differences (*P* < 0.05)

### Protein profiles of alfalfa leaves at different developmental stages

The alfalfa leaves from two developmental stages were assessed by profiling the proteome. Approximately 17,214 unique peptides corresponding to 4540 proteins were successfully identified through LC-MS/MS mass spectrometry identification and a search against the UniProt database employing MASCOT integrated with Proteome Discoverer 1.4 software. A 1.2-fold-change cut-off was used to indicate significant changes in the abundance of differentially expressed proteins (DEPs) during alfalfa development, and 415 proteins were identified. Among these proteins, 256 were down-regulated, and 159 were up-regulated in leaves at the mid-flowering stage compared with the budding stage (Additional file 1).

**Fig. 2:**
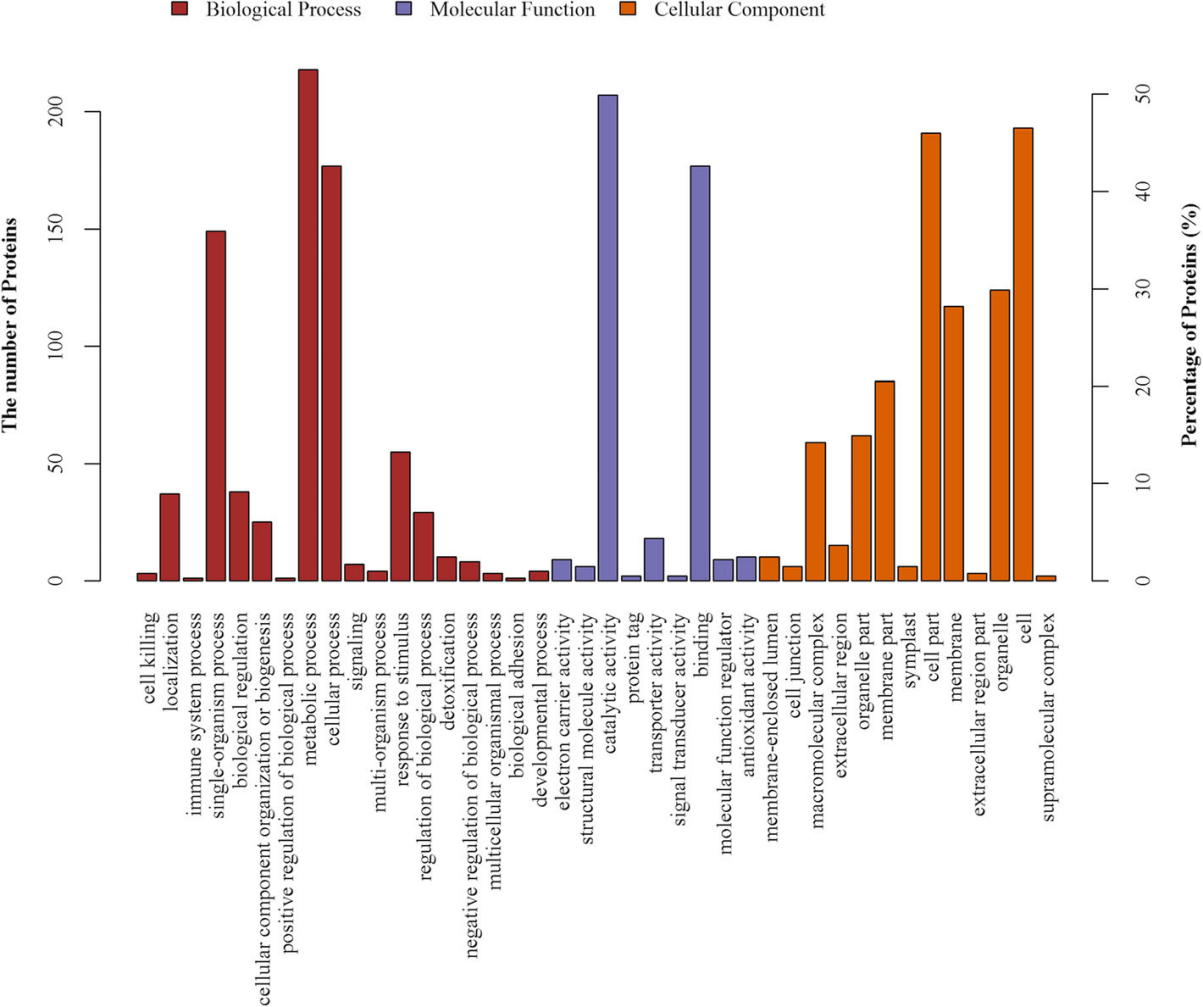
GO classification of the identified proteins. GO classification of the identified proteins during alfalfa flower development. The results are summarized under three main GO categories: biological process (BP), cellular component (CC), and molecular function (MF)

Protein functional analysis using all 415 DEPs based on GO category enrichment was carried out using the GO and UniProt databases. Based on their functional features, the 415 significantly differentially expressed proteins were classified into the biological process (BP), cellular component (CC) and molecular function (MF) categories (Fig. 2). The major functional categories in the BP category were metabolic processes, cellular processes, single-organism processes and response to stimulus. For MF, catalytic activity, binding and transporter activity were the most abundant groups. The cell, organelle and membrane categories were the most abundant groups under CC.

**Fig. 3:**
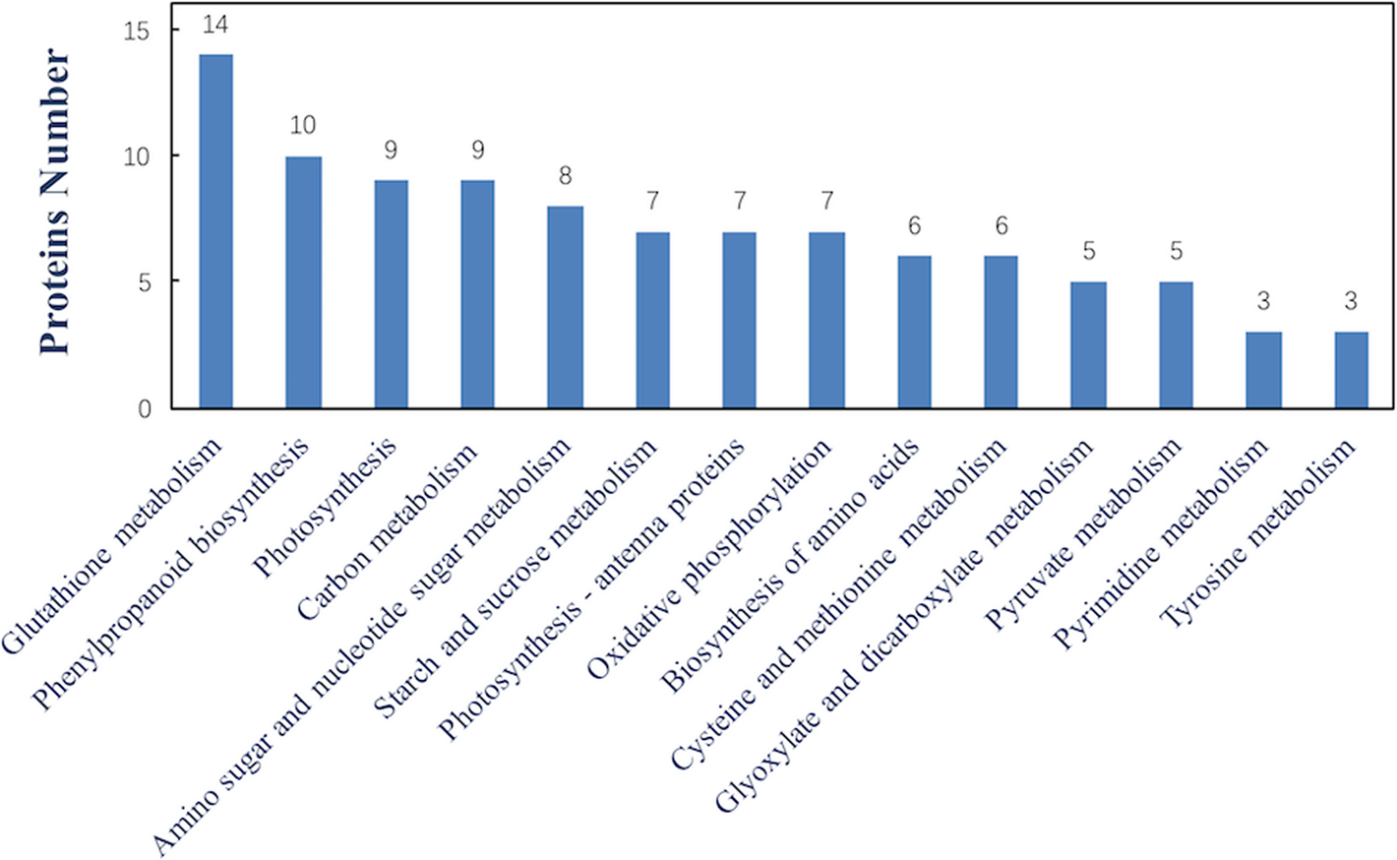
KEGG pathways in which the identified proteins are involved. Signalling pathways of the proteins identified as being involved in the budding to mid-flowering stages of alfalfa development

In addition, KEGG analysis was employed to understand the molecular pathways containing differentially expressed proteins. A total of 415 proteins were assigned to 173 pathways (Additional file 2). These proteins were mainly distributed in glutathione metabolism, phenylpropanoid biosynthesis, photosynthesis, carbon metabolism, amino sugar and nucleotide sugar metabolism, starch and sucrose metabolism, biosynthesis of amino acids, and other categories (Fig. 3). These results indicate that alfalfa nutritional quality related to proteins involved in synthesis and metabolism and the metabolic pathways in which they participate changed.

### Metabolite profiles of alfalfa leaves at different developmental stages

**Fig. 4:**
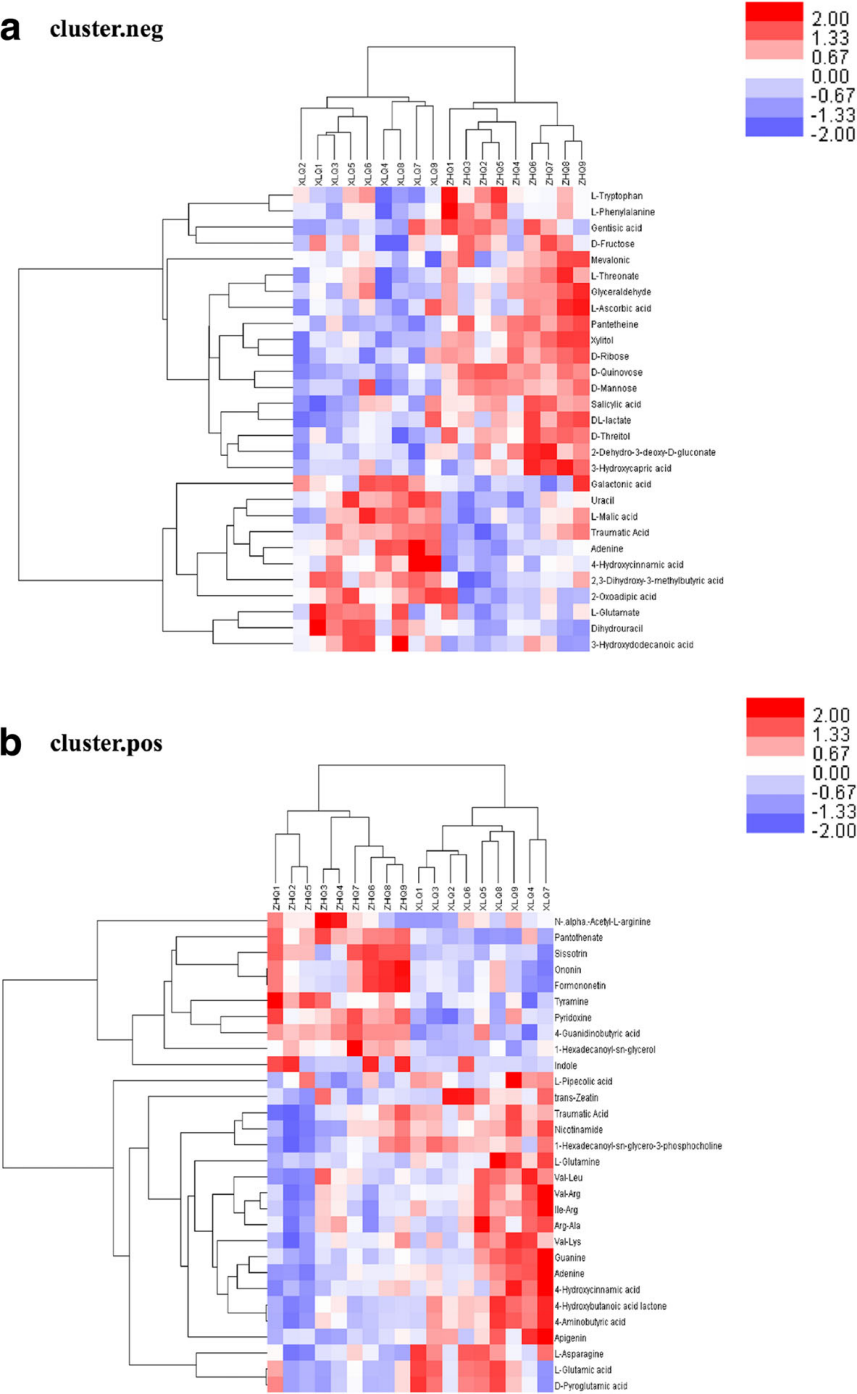
Hierarchical cluster heat map of differential metabolites. Hierarchical cluster heat map of significantly different metabolites during alfalfa leaf development from the budding to the mid-flowering stage. **a** Positive mode, **b** negative mode

**Fig. 5:**
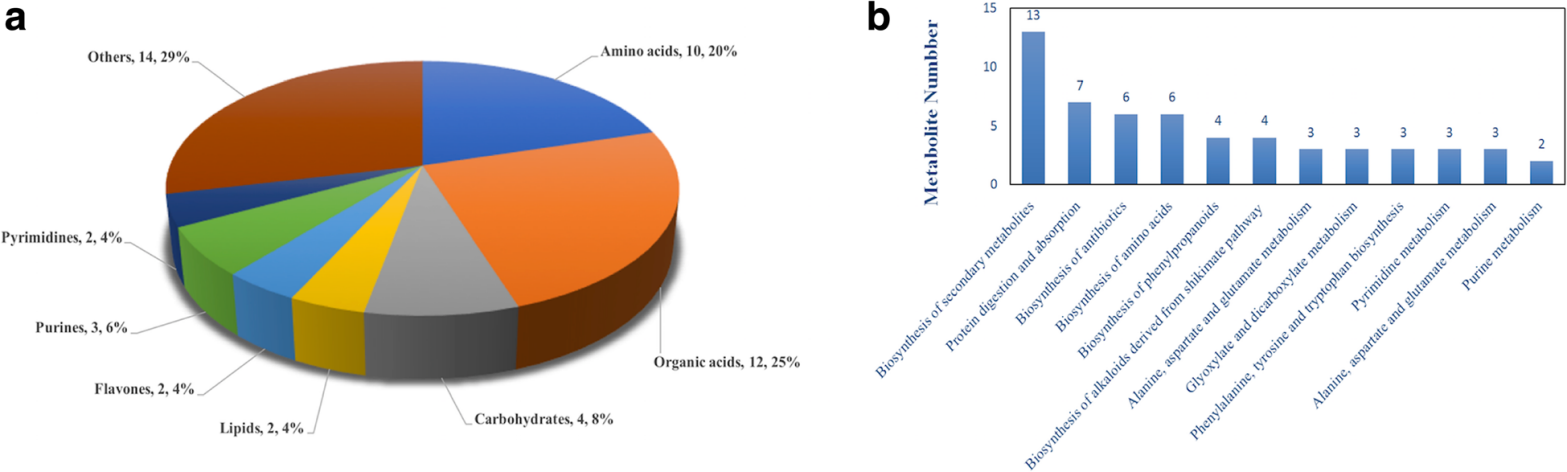
Metabolite classification and KEGG pathway analysis. **a** Classification of the significantly varying metabolites between the two developmental stages. The 49 varying metabolites involved amino acids (10), organic acids (12), carbohydrates (4), purines (3), lipids (2), pyrimidines (2), and others (14). **b** Signalling pathways of the differential metabolites involved in the budding to mid-flowering stages of alfalfa development

The changes in the metabolic profiles of the leaf samples between two groups were analysed using the metabolomics method based on HILIC UHPLC-Q–TOF technology. The significantly different metabolites were selected based on the criteria of an OPLS-DA model VIP > 1 and a *P*-value < 0.05. To evaluate the rationality of the candidate metabolites and more fully and intuitively illustrate the relationship between the samples and the metabolites in samples exhibiting differences in expression patterns, we conducted a hierarchical cluster analysis based on the expression of significantly different metabolites in each group of samples. This approach assisted in the accurate selection of marker metabolites and the investigation of changes in related metabolic processes (Fig. 4). Finally, 49 significant variations in metabolites were detected, which are shown in Table 2. These metabolites mainly included amino acids, organic acids, carbohydrates, purines, lipids and pyrimidines (Fig. 5a). A total of 24 metabolites were up-regulated at flowering, while 25 were down-regulated at flowering. The expression levels of L-glutamic acid, L-asparagine, purine, pyrimidine and other protein synthesis-related metabolites were down-regulated, whereas L-phenylalanine and carbohydrates, lipids and other substances were significantly up-regulated.

**Table 2:** Differential metabolites of leaves between the budding stage and mid-flowering stage

Ionization mode	Metabolite no.	Adduct	RT(s)	m/z	Metabolite	VIP	Fold-change	*P-*value
ESI(+)	1	(M + H-H2O)+	458.83	120.08	Tyramine	2.65	1.47	0.0049
ESI(+)	2	(M + H)+	253.46	447.13	Sissotrin	1.48	3.91	0.0006
ESI(+)	3	(M + H)+	186.58	170.08	Pyridoxine	1.12	1.52	0.0006
ESI(+)	4	(M + H)+	489.09	220.12	Pantothenate	1.58	1.80	0.0000
ESI(+)	5	(M + H)+	190.21	431.13	Ononin	2.70	3.83	0.0196
ESI(+)	6	(M + H)+	190.05	269.08	Formononetin	1.52	3.69	0.0182
ESI(+)	7	(M + H)+	646.43	146.09	4-Guanidinobutyric acid	1.12	1.37	0.0000
ESI(+)	8	(M + H-H2O)+	285.03	313.27	1-Hexadecanoyl-sn-glycerol	1.41	1.50	0.0260
ESI(−)	9	(M-H)-	377.88	151.06	Xylitol	3.23	1.56	0.0001
ESI(−)	10	(M-H)-	62.82	137.02	Salicylic acid	3.66	2.29	0.0109
ESI(−)	11	(M-H)-	402.66	277.12	Pantetheine	1.19	2.47	0.0000
ESI(−)	12	(M + CH3COO)-	265.12	207.09	Mevalonic acid	2.00	1.88	0.0139
ESI(−)	13	(M-H)-	458.41	203.08	L-Tryptophan	2.33	1.74	0.0265
ESI(−)	14	(M-H)-	133.56	135.03	L-Threonate	1.06	1.30	0.0042
ESI(−)	15	(M-H)-	456.39	164.07	L-Phenylalanine	1.63	1.34	0.0143
ESI(−)	16	(M-H)-	134.08	89.02	Glyceraldehyde	1.32	1.23	0.0136
ESI(−)	17	(M-H)-	117.76	153.02	Gentisic acid	2.17	1.68	0.0364
ESI(−)	18	(M + CH3COO)-	539.79	181.07	D-Threitol	1.31	1.69	0.0001
ESI(−)	19	(M + CH3COO)-	486.17	209.07	D-Ribose	1.16	1.48	0.0001
ESI(−)	20	(M + CH3COO)-	465.33	223.08	D-Quinovose	3.74	2.29	0.0000
ESI(−)	21	(M-H)-	276.68	179.06	D-Mannose	1.77	1.58	0.0009
ESI(−)	22	(M-H)-	550.77	89.02	DL-lactate	2.00	1.19	0.0070
ESI(−)	23	(M + Na-2H)-	173.53	209.12	3-Hydroxycapric acid	1.72	1.56	0.0221
ESI(−)	24	(M + CH3COO)-	673.63	237.06	2-Dehydro-3-deoxy-D-gluconate	2.37	1.34	0.0030
ESI(+)	25	(M + H)+	696.52	246.18	Val-Lys	1.15	0.72	0.0362
ESI(+)	26	(M + H)+	656.61	274.19	Val-Arg	2.16	0.67	0.0061
ESI(+)	27	(M + CH3CN + H)+	79.12	261.15	trans-Zeatin	1.39	0.35	0.0487
ESI(+)	28	(M + H)+	105.57	123.06	Nicotinamide	7.09	0.61	0.0243
ESI(+)	29	(M + H)+	684.47	147.08	L-Glutamine	1.70	0.76	0.0159
ESI(+)	30	(M + H)+	730.92	148.06	L-Glutamic acid	2.26	0.79	0.0130
ESI(+)	31	(M + H)+	692.74	133.06	L-Asparagine	2.27	0.58	0.0017
ESI(+)	32	(M + H)+	613.65	288.20	Ile-Arg	2.19	0.66	0.0034
ESI(+)	33	(M + H)+	402.69	152.06	Guanine	2.20	0.56	0.0164
ESI(+)	34	(M + H)+	730.92	130.05	D-Pyroglutamic acid	1.95	0.81	0.0193
ESI(+)	35	(M + H)+	71.69	271.06	Apigenin	6.28	0.30	0.0265
ESI(+)	36	(M + H)+	677.16	87.04	4-Hydroxybutanoic acid lactone	2.90	0.81	0.0002
ESI(+)	37	(M + H)+	677.15	104.07	4-Aminobutyric acid	2.58	0.82	0.0004
ESI(+)	38	(M + H)+	338.63	496.34	1-Hexadecanoyl-sn-glycero-3-phosphocholine	2.17	0.68	0.0271
ESI(−)	39	(M-H)-	145.45	111.02	Uracil	1.77	0.49	0.0010
ESI(−)	40	(M-H)-	534.46	227.13	Traumatic Acid	2.23	0.63	0.0166
ESI(−)	41	(M-H)-	796.59	133.01	L-Malic acid	1.34	0.87	0.0327
ESI(−)	42	(M-H)-	730.88	146.05	L-Glutamate	2.28	0.83	0.0483
ESI(−)	43	(M-H)-	781.63	195.05	Galactonic acid	1.13	0.61	0.0423
ESI(−)	44	(M-H)-	277.29	134.05	Adenine	11.09	0.63	0.0010
ESI(−)	45	(M-H)-	688.71	113.04	Dihydrouracil	2.37	0.52	0.0021
ESI(−)	46	(M-H)-	595.57	163.04	4-Hydroxycinnamic acid	1.39	0.65	0.0077
ESI(−)	47	(M + Na-2H)-	113.11	237.15	3-Hydroxydodecanoic acid	1.44	0.48	0.0129
ESI(−)	48	(M-H2O-H)-	558.25	141.02	2-Oxoadipic acid	1.29	0.83	0.0063
ESI(−)	49	(M-H)-	202.24	133.05	2,3-Dihydroxy-3-methylbutyric acid	3.57	0.40	0.0014

The fold-change (FC) was calculated as the average metabolite concentration in the leaves during the mid-flowering stage relative to the budding stage

We submitted the differential metabolites to the KEGG website for the analysis of relevant pathways, and we found that these differentially expressed metabolites were mainly involved in the biosynthesis of secondary metabolites, protein digestion and absorption, the biosynthesis of amino acids and the biosynthesis of phenylpropanoids (Fig. 5b). The changes in these metabolites provide important information for our study of changes in the nutritional quality of alfalfa.

### Proteome-metabolome data co-analysis

**Fig. 6:**
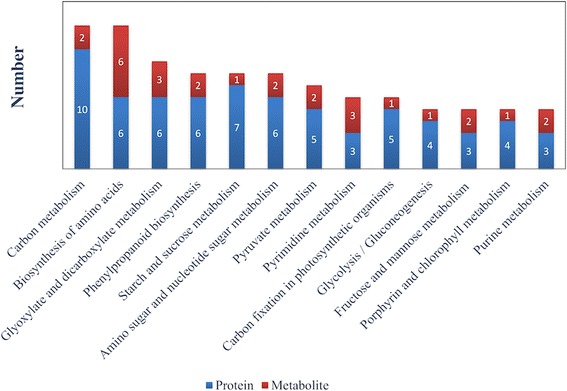
Common pathways of candidate proteins and metabolites. Signalling pathways of the proteins and metabolites commonly involved in the budding to mid-flowering stages of alfalfa development. Blue represents the number of differentially expressed proteins. Red represents the number of differentially abundant metabolites

To associate the results of proteomics and metabolomics analyses, we chose metabolic pathways as the carrier and conducted a mapping analysis based on the differences in proteins and metabolites. In total, matches to 57 metabolic pathways showed changes (Additional file 3). Further analysis of these differential metabolic pathways revealed that they were mainly involved in energy metabolism. We further selected the metabolic pathways related to nutritional metabolism associated with alfalfa, the biosynthesis of amino acids, phenylpropanoid biosynthesis and starch and sucrose metabolism, among others (Fig. 6).

**Fig. 7:**
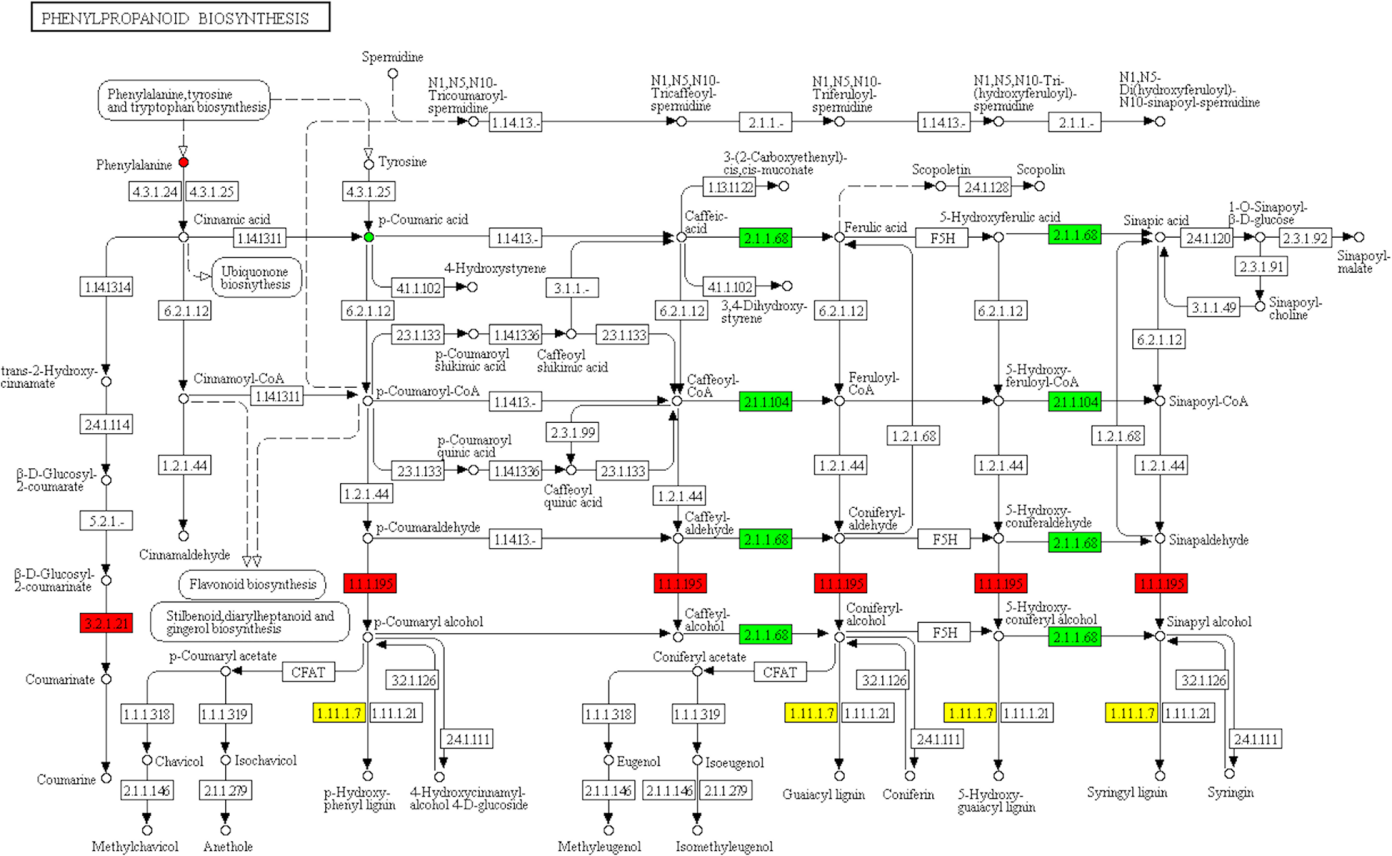
Phenylpropanoid biosynthesis pathway. Proteins (□) and metabolites (o) with differential abundance involved in phenylpropanoid biosynthesis metabolism were mapped to the corresponding metabolic pathways in KEGG. Red indicates up-regulation; green indicates down-regulation; and yellow indicates no significant difference. The up-regulated proteins were beta-glucosidase [EC: 3.2.1.21] and cinnamyl-alcohol dehydrogenase [EC: 1.1.1.195], and the down-regulated proteins in this pathway were caffeic acid 3-O-methyltransferase [EC: 2.1.1.68] and caffeoyl-CoA O-methyltransferase [EC: 2.1.1.104]. The up-regulated metabolite was L-phenylalanine, and the down-regulated metabolite was 4-hydroxycinnamic acid

Following annotation, phenylpropanoid biosynthesis was sequentially mapped to metabolic pathways (Fig. 7). The proteins and metabolites with differential abundances were clearly mapped primarily to the phenylpropanoid biosynthesis pathway in KEGG. Proteins and metabolites involved in phenylpropanoid biosynthesis were quite active from budding to mid-flowering, among which L-phenylalanine, beta-glucosidase [EC:3.2.1.21], and cinnamyl-alcohol dehydrogenase [EC:1.1.1.195] were up-regulated, and 4-hydroxycinnamic acid, caffeic acid 3-O-methyltransferase [EC:2.1.1.68], and caffeoyl-CoA O-methyltransferase [EC:2.1.1.104] were down-regulated.

## Discussion

In omics studies, proteomics and metabolomics are later additions involving sequential events and fulfilling the understanding of the functional aspects of the living system. Proteomics mainly delineates the expression profiles of the proteins, including the analysis of protein structure, and reveals genetic modifications. In contrast, metabolomics refers to the complete analysis of low-molecular-weight compounds. Metabolomics plays a vital role in determining the changes in the compounds under different conditions or the changes over time and can be employed to reveal the biochemical compositional characteristics, such as colour and smell, of bioactive compounds; these characteristics are not possible to predict by external sensing [28]. As stated by Gahlaut et al. [29], the complete elucidation of biological responses at different stages can be compiled at the transcriptome, proteome and metabolome levels, and proteomics reflects modulations in the metabolomics. Carrari et al. [30] performed a metabolomics analysis to discriminate the developmental stages of tomato fruit. These findings indicate the necessity of addressing the integration of proteomics and metabolomics in the present study. More importantly, the nutritional changes in the study plant ‘alfalfa’ obtained good support from the analysis of proteomics and metabolomics integration. This integration offers a well-detailed comprehensive molecular picture during the transition of budding to the mid-flowering stages in the alfalfa plant. A recent study by Akpunarlieva [31] provided further anchorage to the current study; in that study, researchers integrated proteomics and metabolomics to identify the molecular changes in the living system.

### Effect of the growth period on the nutritional quality of alfalfa

Crude protein is the general term for nitrogen compounds in feed and is the main decision indicator for RFV. J.F.S. Lamb et al. [32] reported that the crude protein content of alfalfa leaves continuously decreased with delayed harvest maturity. Previous reports have indicated that alfalfa proteins are mainly located in chloroplasts, and the transition from vegetative growth to reproductive growth of alfalfa increases nutrient production and lignification, leading to a decrease in the crude protein content [33]. This observation agrees with our results.

The content of NDF in feed is negatively correlated with the feed intake of dry matter, and the content of ADF directly affects the digestibility of forage. These findings are due to factors related both to the plant itself and to human factors. First, the alfalfa harvest time is delayed, and the drying time is too long, which will cause serious lignification of alfalfa. Additionally, the values of the NDF and ADF indicators will be high, resulting in reduced NDF digestibility and a reduced RFV of alfalfa. Second, it is difficult to guarantee uniformity of planting and harvesting times, resulting differences in the maturity and quality of alfalfa. Third, a low degree of mechanization results in long harvest, drying and packing times. Not only will these factors cause increased lignification, but many leaves will fall off, resulting in a lower crude protein content.

### Changes in ADF and NDF contents

#### Carbohydrate metabolism and hemicellulose synthesis

Hemicellulose is a heterogeneous multimer consisting of several different types of monosaccharides. These sugars are pentoses and hexoses, including xylose, arabinose, mannose and galactose, among others. The main glycosyl groups constituting hemicellulose are the D-xylosyl, D-mannosyl, D-glucosyl, D-galactosyl, L-arabinosyl, 4-O-methyl-D-glucuronic acid-based, D-galacturonic acid and D-glucuronic acid groups. Our results (Table 3) show that D-mannose is up-regulated at mid-flowering, as are alpha-glucosidase (G7JXA7) and alpha-amylase (A0A072U233), which can hydrolyse starch to produce D-glucose.

**Table 3:** Carbohydrate metabolism-related proteins and metabolites

Proteins	Accession	Description	Coverage	Fold-change	*P-*value
	A0A072UTK4	Glycoside hydrolase family 1 protein	9.15	1.25	0.0053
	G7J6L9	Glycoside hydrolase family 3 protein	6.54	1.37	0.0115
	G7KWW4	Pectinesterase	3.44	1.37	0.0126
	A5JTQ3	Beta-xylosidase/alpha-L-arabinofuranosidase 2	14.99	1.73	0.0221
	G7JUT0	UDP-glucuronic acid decarboxylase	22.81	1.34	0.0224
	G8A1F4	Glycosyltransferase	1.84	1.32	0.0240
	A0A072UKS2	PfkB family carbohydrate kinase	21.15	0.80	0.0127
Metabolites	Metabolite	Adduct	VIP	Fold-change	*P-*value
	D-Ribose	(M + CH3COO)-	1.16	1.48	0.0001
	D-Quinovose	(M + CH3COO)-	3.74	2.29	0.0000
	D-Mannose	(M-H)-	1.77	1.58	0.0009

Simultaneously, we noticed a significantly higher abundance of UDP-glucuronic acid decarboxylase (UXS) (G7JUT0), which can catalyse UDP-glucuronic acid to produce xylan [34]. Xylan is the main component of hemicellulose in plants. Therefore, we believe that the hemicellulose content of alfalfa leaves during the flowering period is higher than that in the budding period. NDF includes cellulose, hemicellulose, lignin, silica, keratin and wax, and ADF includes cellulose, lignin and acid insoluble ash [35], thus explaining why the increase in NDF content indicated by our results is significantly higher than that for ADF.

#### Phenylpropanyl biosynthesis pathway and lignin synthesis

Lignin is one of the components of ADF and NDF and is an important factor affecting the digestibility of herbage, where a higher lignin content results in lower digestibility [36]. The biosynthesis of lignin begins with phenylalanine [37]. After a series of hydroxylation, methylation, ligation and reduction reactions to produce monomers, the monomers are further oxidized to produce the corresponding lignin. Cinnamyl-alcohol dehydrogenase (CAD) is one of the key enzymes in lignin synthesis and can catalyse a variety of cinnamaldehyde components to produce lignin monomer precursors; therefore, CAD was the first enzyme to be studied in the lignin synthesis pathway [38]. Alfalfa leaves in the mid-flowering stage exhibit significantly higher abundance of L-phenylalanine and higher abundance of CAD compared with the budding stage (Table 2, Fig. 7). An increase in the biosynthesis of amino acid results in an increased abundance of phenylalanine, which provides the starting material for lignin synthesis. At the same time, CAD expression is up-regulated. CAD participates in the reduction reaction of the last step of lignin monomer synthesis. When CAD activity is decreased, the lignin content is reduced, and the number of G and S monomers, which contain pineal aldehyde and mustard aldehyde, is significantly lower [39–41]

Thus, the up-regulation of CAD abundance is conducive to lignin synthesis. We infer that alfalfa at the mid-flowering stage is characterized by more active phenylpropanoid biosynthesis pathways, which may promote the synthesis of lignin. Finally, the reaction of alfalfa NDF and ADF contents increases, and the nutritional quality of alfalfa decreases.

### Crude protein content changes

#### Amino acid synthesis and metabolism

The crude protein content of alfalfa is usually between 15 and 22% and is mainly distributed in the leaves. The crude protein is divided into two major categories: true protein and non-protein nitrogen (NPN), according to chemical properties. Non-protein nitrogen includes free amino acids, amides, purines, pyrimidines and alkaloids, accounting for approximately 1/3 of the total nitrogen content of alfalfa. L-glutamic acid and glutamine are the tectonic units of the protein. They play a vital role in nitrogen metabolism and are synthetic precursors of a variety of amino acids, purines and pyrimidines in the organism [42, 43]. Previous studies [44–46] in wheat have shown that the contents of L-glutamic acid and protein are significantly or extremely significantly positively correlated. Our results (Table 4) show that the content of L-glutamic acid at mid-flowering is significantly lower than in the budding period. At the same time, the contents of L-glutamine, L-asparagine, guanine, adenine, uracil, and dihydrouracil are also lower, which may be one of the reasons for the decrease in protein content.

**Table 4:** Amino acid synthesis-related proteins and metabolites

Proteins	Accession	Description	Coverage	Fold-change	*P*-value
	Q6J9X7	Chloroplast cystathionine beta lyase	11.65	1.26	0.0084
	G7L3W1	S-adenosylmethionine synthase	28.97	1.53	0.0259
	A0A072VKL5	S-adenosylmethionine synthase	33.59	1.28	0.0277
	G7JCK0	Ketol-acid reductoisomerase	23.45	1.23	0.0329
	A0A072UXL1	Deoxyuridine 5′-triphosphate nucleotidohydrolase	11.33	1.29	0.0347
	Q9SPM6	Nod factor binding lectin-nucleotide phosphohydrolase	9.23	2.65	0.0218
	G7ZV13	ATP sulfurylase	18.31	1.50	0.0318
	A0A072W0C8	ATP sulfurylase	14.19	1.51	0.0331
	G7KXR2	Transaldolase family protein	24.15	0.82	0.0007
	A0A072TNP6	4-hydroxy-tetrahydrodipicolinate synthase	5.25	0.80	0.0031
	G7LF25	RNA polymerase II, Rpb4, core protein	6.52	0.74	0.0073
Metabolites	Metabolite	Adduct	VIP	Fold-change	*P*-value
	L-Tryptophan	(M-H)-	2.33	1.74	0.0265
	L-Threonate	(M-H)-	1.06	1.3	0.0042
	L-Phenylalanine	(M-H)-	1.63	1.34	0.0143
	Val-Lys	(M + H)+	1.15	0.72	0.0362
	Val-Arg	(M + H)+	2.16	0.67	0.0061
	L-Glutamine	(M + H)+	1.70	0.76	0.0159
	L-Glutamic acid	(M + H)+	2.26	0.79	0.013
	L-Asparagine	(M + H)+	2.27	0.58	0.0017
	Ile-Arg	(M + H)+	2.19	0.66	0.0034
	Guanine	(M + H)+	2.2	0.56	0.0164
	D-Pyroglutamic acid	(M + H)+	1.95	0.81	0.0193
	Uracil	(M-H)-	1.77	0.49	0.001
	Dihydrouracil	(M-H)-	2.37	0.52	0.0021

Interestingly, we found that L-tyrosine and L-phenylalanine were up-regulated. However, the results showed that no related proteins exhibited changes; therefore, we speculate that this up-regulation may result from protein hydrolysis, which may be one of the causes of the reduced albumin crude protein content. L-tyrosine can be produced via the hydroxylation of phenylalanine [47] and may participate with phenylalanine in plant glucose metabolism and fat metabolism. This speculation is consistent with the results of our test showing that sugar and fat metabolism was increased, while carbohydrate and lipid metabolites were increased.

A recent study performed by Ullah et al. [48] provided a thorough comparison among different Triticeae species by profiling the metabolites of roots and leaves under drought stress. By correlating the morphology of plant species with length, surface area and root diameter via metabolomics, this study provided concrete support to the present study. Another study by the same group to identify proteome changes in the wheat plant clearly indicated post-translational modifications, giving further support for utilizing omics analyses in plant systems [49]. Interestingly, the metabolites (phenylalanine, mannose, valine, lysine, asparagine, aminobutyric acid, malic acid, glutamate and glyceraldehyde) found in the present study matched those identified in the above investigations. Further, the experimental studies and discussions in these reports strengthen our findings with the alfalfa plant using proteomics integrated with metabolomics.

#### Photosynthesis and chlorophyll metabolism

Approximately 30 to 50% of the protein in alfalfa leaves is present in the chloroplasts. Research on plant physiology has shown that the starting material for the synthesis of chlorophyll is glutamic acid or a-ketoglutaric acid [50]. Probably, a decrease in glutamic acid content will inhibit the synthesis of chlorophyll and inhibit photosynthesis.

Our results showed that most of the light-harvesting chlorophyll protein complexes (LHCs) in the photosystem in the photosynthesis pathway (lhca1, lhca3, lhcb1, lhcb2, lhcb3, lhcb4, and lhcb6) were down-regulated (Additional file 4), as was the glutamic acid content and that of ATP synthase (B7FH20), the key enzyme in ATP synthesis, which can provide energy and play an important role in photosynthesis [51] (Table 5). These changes inhibit photosynthesis and further limit the synthesis of chlorophyll and chloroplast activity, resulting in a decrease in protein content. Our study complements the results by Aranjuelo et al. [52] who studied the physiological, metabolic and proteomic processes active during photosynthetic inhibition in the alfalfa plant. Specifically, reductions in glutamic acid and asparagine (Table 4) were found in both studies. Further, the overall protein and ATP synthase levels were affected, leading to down-regulation. These consistencies with the alfalfa plant used in the present study and the photosynthetic analysis by Ajanjuelo et al. support the compatibility of metabolomics with conditional changes in plant growth.

**Table 5:** Photosynthesis and chlorophyll metabolism-related proteins and metabolites

Proteins	Accession	Description	Coverage	Fold-change	*P-*value
	I3SIG9	Chlorophyll a-b binding protein, chloroplastic	60.15	0.83	0.0059
	B7FHZ5	Chlorophyll a-b binding protein, chloroplastic	55.85	0.77	0.0074
	A0A072U7I8	Chlorophyll a-b binding protein, chloroplastic	19.42	0.68	0.0111
	G7INT9	Chlorophyll a-b binding protein, chloroplastic	46.36	0.72	0.0114
	I3SZG9	Chlorophyll a-b binding protein, chloroplastic	37.91	0.80	0.0220
	B7FIZ5	Chlorophyll a-b binding protein, chloroplastic	21.8	0.75	0.0222
	A0A072VAK2	Chlorophyll a-b binding protein, chloroplastic	44.3	0.81	0.0432
	B7FH20	ATP synthase	44.67	0.75	0.0014
Metabolites	Metabolite	Adduct	VIP	Fold-change	*P-*value
	L-Glutamine	(M + H)+	1.70	0.76	0.0159
	L-Glutamic acid	(M + H)+	2.26	0.79	0.0130

## Conclusions

Our data highlight the metabolic and protein changes occurring in the leaves of alfalfa and reveal complex metabolic changes from the budding stage to the mid-flowering stage. Large numbers of differentially expressed metabolites and differentially expressed proteins were found to be mainly involved in carbohydrate metabolism, starch and sucrose metabolism, phenylpropanoid biosynthesis and the biosynthesis of amino acids. Alfalfa leaves in the mid-flowering stage contain less crude protein, due to a decrease in L-glutamic acid content. The metabolism of carbohydrates provides the raw material for the synthesis of hemicellulose, resulting in an increase in the hemicellulose content of alfalfa leaves, further leading to an increase in the NDF content. In addition, the increase in L-phenylalanine content provides the conditions required for lignin synthesis. These are the main factors leading to the decline in alfalfa RFV and quality. In summary, these results present an overview of the protein and metabolic processes operating in alfalfa leaves at the budding and mid-flowering stages. This study provides innovative and in-depth results elucidating the differences between alfalfa at these two important stages. Our findings also indicate the reasons for these changes. Hence, the relationships between the reduction in the nutritional value of alfalfa and complex biological processes have been elucidated in this study, providing a theoretical basis for of the production of high-quality alfalfa hay and guiding future research.

## Additional files

Additional file 1: **Table S1.** Protein quantification and analysis of significant differences. (XLSX 81 kb)

Additional file 2: **Table S2.** KEGG pathways in which differentially expressed proteins are involved. (XLSX 23 kb)

Additional file 3: **Table S3.** Proteome-metabolome data co-analysis. (XLSX 13 kb)

Additional file 4: **Figure S1.** Differential expression of the light-harvesting chlorophyll protein complex (LHC) in the photosystem in the photosynthesis pathway. (TIFF 433 kb)

## Abbreviations

ADF: Neutral detergent fibre
CAD: Cinnamyl-alcohol dehydrogenase
CP: Crude protein
DMI: Dry matter intake
FC: Fold-change
LHC: Light-harvesting chlorophyll protein complex
NDF: Neutral detergent fibre
NPN: Non-protein nitrogen
OPLS-DA: Orthogonal partial least-squares discriminant analysis
PCA: Principal component analysis
RFV: Relative feed value
TMT: Tandem mass tag
UXS: UDP-glucuronic acid decarboxylase
VIP: Variable influence on projection
