# Supplementary figures and images for: Proteomics integrated with metabolomics: analysis of the internal causes of nutrient changes in alfalfa at different growth stages
Source: BMC Plant Biol. 2018 May 4;18:78. doi: 10.1186/s12870-018-1291-8 (PMC5935980; doi:10.1186/s12870-018-1291-8)

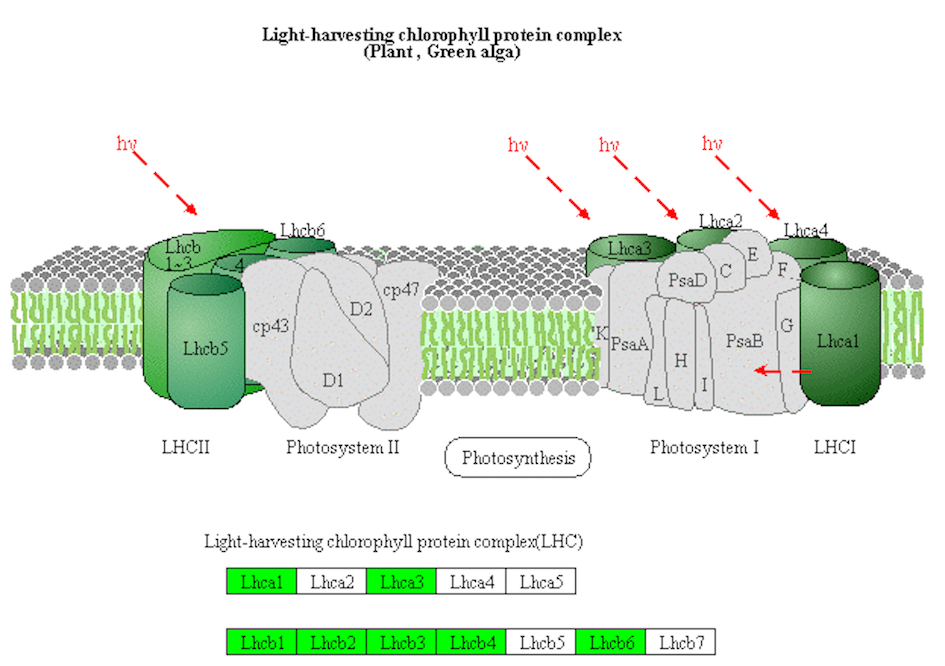

Supplement: Supplementary file 4 — Figure S1. Differential expression of the light-harvesting chlorophyll protein complex (LHC) in the photosystem in the photosynthesis pathway. (TIFF 433 kb) [file 12870_2018_1291_MOESM4_ESM.tiff]
